# The contribution of international health volunteers to the health workforce in sub-Saharan Africa

**DOI:** 10.1186/1478-4491-5-19

**Published:** 2007-07-31

**Authors:** Geert Laleman, Guy Kegels, Bruno Marchal, Dirk Van der Roost, Isa Bogaert, Wim Van Damme

**Affiliations:** 1 Department of Public Health, Institute of Tropical Medicine, Antwerp, Belgium

## Abstract

**Background:**

In this paper, we aim to quantify the contribution of international health volunteers to the health workforce in sub-Saharan Africa and to explore the perceptions of health service managers regarding these volunteers.

**Methods:**

Rapid survey among organizations sending international health volunteers and group discussions with experienced medical officers from sub-Saharan African countries.

**Results:**

We contacted 13 volunteer organizations having more than 10 full-time equivalent international health volunteers in sub-Saharan Africa and estimated that they employed together 2072 full-time equivalent international health volunteers in 2005. The numbers sent by secular non-governmental organizations (NGOs) is growing, while the number sent by development NGOs, including faith-based organizations, is mostly decreasing. The cost is estimated at between US$36 000 and US$50 000 per expatriate volunteer per year. There are trends towards more employment of international health volunteers from low-income countries and of national medical staff.

Country experts express more negative views about international health volunteers than positive ones. They see them as increasingly paradoxical in view of the existence of urban unemployed doctors and nurses in most countries. Creating conditions for employment and training of national staff is strongly favoured as an alternative. Only in exceptional circumstances is sending international health volunteers viewed as a defendable temporary measure.

**Conclusion:**

We estimate that not more than 5000 full-time equivalent international health volunteers were working in sub-Saharan Africa in 2005, of which not more than 1500 were doctors. A distinction should be made between (1) secular medical humanitarian NGOs, (2)development NGOs, and (3) volunteer organizations, as Voluntary Service Overseas (VSO) and United Nations volunteers (UNV). They have different views, undergo different trends and are differently appreciated by government officials.

International health volunteers contribute relatively small numbers to the health workforce in sub-Saharan Africa, and it seems unlikely that they will do more in the future. In areas where they play a role, their contribution to service delivery is sometimes very significant.

## Background

The human resource crisis is particularly acute in sub-Saharan Africa. WHO defined 57 countries as having a critical shortage, and 36 of them are in sub-Saharan Africa [1]. The reasons for this have to do with intake (training, recruitment etc), stock management (productivity, motivation, quality, ...) and outflow (attrition, retention, migration issues, ...). Response to human resource problems – particularly those related to income and performance – is often piecemeal and improvised. Although most commentators agree that strategies have to be combined to address the different dimensions of this complex global problem, few countries propose structural responses other than decentralization [2].

One of the options that has been touted in recent years is to send professionals from industrialized countries to make up for the scarcity of health workers in poor countries, making the most of the willingness of (young) professionals from these countries to integrate a period of work overseas within their career plan. Preparatory work for the U.S. President's Emergency Plan for AIDS Relief (PEPFAR), for example, refers to such 'international volunteers' as a way to make up for the lack of qualified human resources for health (HRH) to implement HIV/AIDS programs [3].

Employed by non-governmental organizations (NGOs) based in the north, these international volunteers often play a highly visible role [4]. However, virtually nothing has been published on numbers, cost and impact of these expatriate staff on health systems and health care delivery. In the first part of this paper, we set out to quantify the contribution of international health volunteers. Second, we explore the perceptions of both the sending organizations and health service managers from the south regarding the role of international health volunteers. Finally, we identify factors of successful contribution of international health volunteers to health services in the south.

## Methods

In this study, we define 'international volunteers' operationally as expatriate employees of non-for-profit NGOs based in the North but with field activities in sub-Saharan Africa. This excludes local employees of international NGOs, as well as international civil servants, technical assistants employed by bilateral donors or their implementation agencies, private consultancy companies, or international medical staff recruited by governments. These international volunteers are characterized by the commitment that is part of the institutional culture of their employing organizations, by the fact that they are often relatively young and employed under relatively modest salary conditions. The quantitative analysis is focused mainly on European NGOs and United Nations Volunteers (UNV).

Data were collected from various sources. First, Google and Medline searches (keywords: NGO, PVO, volunteers 'UN volunteers', and 'volunteers and health') provided the initial information that was used to identify the sending organizations. From there, preliminary data on numbers, characteristics and profile of volunteers was collected from the websites. As a result of additional snowballing, 13 organizations sending more than 10 volunteers were identified. In a second phase, this information was complemented through e-mail surveys and telephone interviews of the human resource managers of the concerned organizations. Information collected through the survey included numbers of employees overseas (point prevalence on 1/1/05 and trends), qualifications, geographical distribution, type of work and costs of deployment to the NGO, and difficulties and challenges in recruitment and employment. The interviews provided insights in the perceptions of the organization regarding the role and contribution of their international health volunteers.

In a third phase, we conducted two group discussions with 8 experienced medical officers from sub-Saharan African countries. The participants were drawn from the students of the international master in public health of the Institute of Tropical Medicine, Antwerp, and were all experienced health service managers in the public and private not-for-profit sector. The discussions focused on their perceptions of the effects and usefulness of the deployment of international health volunteers in their work setting. More specifically themes included strengths and weaknesses of international health volunteers, possible alternatives and conditions under which international health volunteers could make optimal contributions. The discussions were moderated by one researcher and notes taken during the discussion by another.

To our knowledge, no analytical framework for studying the contribution of international health volunteers has been published. Given the explorative nature, we set out with a simple framework for the quantitative analysis. It makes the distinction between types of sending organization (medical organizations, emergency versus development organizations, ...), number of staff sent out, qualifications (medical: doctor, nurse, other; and non-medical), kind of work carried out by the volunteers (clinical service provision, management, policy advice, training) and duration of deployment. Additional information on numbers of staff sent out and cost was then linked to this framework. This allowed us to identify some trends and compare between types of organizations. For this, we make the distinction between operational organizations and umbrella organizations that unite and represent national operational branches.

## Results

In this section we first present the findings of the surveys, the telephone interviews with HR managers of sending organizations, and the discussion group findings.

### Results of the surveys

Table 1 gives an overview of a number of features of international health volunteers employed by the organizations that were surveyed. It should be noted that some medical NGOs send staff on short-term assignments. For such organizations, our notion of "prevalence of international health volunteers on 1 January 2005" did not make much sense. They could only report on the number of volunteers sent per year. In the table these numbers are reported between brackets.

**Table 1:** Expatriate health volunteers working overseas with volunteer organizations*

	Expatriate health volunteers				Sub-Saharan Africa		Clinical work	Other, such as management education policy making	Comments
				
Organization	Total	Doctors	Nurses	Other	%	Total number of medical staff (full-time equivalent)			
Médecins sans Frontières (all sections)	2026	27%	30%	43%	60%	1216	60%	40%	40% non – medical ('public health technicians').
Voluntary Service Overseas (UK)	215	16%	14%	70%	78%	168	20%		Worldwide: 1382 VSO volunteers.
United Nations Volunteers	400	51%	16%	33%	38%	152			>5600 skilled professionals per year
Oxfam International GB	272				53%	145		100%	Organization is not a medical NGO, data refer to health advisers, promoters.
Handicap International (France + Belgium)	179			100%		118		100%	Almost all staff are paramedic (kine, ortho, psychologists).
Medici con l'Africa CUAMM (Italy)	84	67%	10%	23%		84	30–40%	60–70%	
Médecins du Monde (France)	149	30%	20%	50%	34%	51	50%	50%	
Action contre la Faim (France)	42	19%	81%	0%	79%	33	0%	100%	
Doctors without Vacation (Belgium)	(400)^†^	46%	44%	10%	100%	30	100%		Exclusively short term missions (2 to 4 weeks).
Christian Blind Mission (all sections)	(116)^†^	37%	3%	60%	64%	25	53%	47%	Many short time missions.
World Vision (Europe)	24				100%	24		100%	
Cordaid (Netherlands)	35	50%	25%	25%	40%	14	75%	25%	
Save the Children (UK)	12	50%	50%			12		100%	Advisors, programme managers.
TOTAL						2072			

* Estimates for 1 January 2005 (only organizations employing more than 10 full-time equivalent expatriate health volunteers in sub-Saharan Africa are reported).

†: number dominated by many short mission

### Numbers of staff deployed

With our survey among volunteer organizations from the North, we could document that the larger organizations together employed at any point in time in 2005 around 2072 international health volunteers in sub-Saharan Africa.

### Duration of deployment

Strikingly, most international health volunteers spend less than two years in one particular setting. The length of 'short' missions ranges from as short as 2 or 3 weeks to as long as 2 years. For organizations working in relief, short missions are mostly for emergency operations. For those working exclusively in development assistance, short missions are carried out by consultants to perform elective surgery or bedside teaching. Relatively few international health volunteers are contracted for assignments of more than 2 years.

### Qualifications of staff

Regarding qualifications, there is quite some variety, in function of the mission and work carried out by the organization. Handicap International, for example, sends no doctors or nurses, while for 5 other organizations, doctors make up more than half of their deployed workforce.

### Type of work

Between 50 to 60% of international health volunteers carry out clinical work; the others are engaged in a variety of other functions, ranging from management or training to policy work.

### Type of organizations

The northern volunteer organizations that send international health volunteers can roughly be divided into three categories: (1) secular medical NGOs, such as *Médecins Sans Frontières*, which often identify themselves as humanitarian organizations; (2) development NGOs, often rooted in Christian missionary organizations, but including also a number of secular NGOs that are mainly involved in long-term development aid; & (3) volunteer organizations which define sending volunteers as their core mission, such as Voluntary Service Overseas (VSO) or United Nations Volunteers (UNV). The newly created US Global Health Service Corps [3] also fits in this third category.

### Trends in deployment: from substitution to empowerment, from expatriate to national staff

Against a backdrop of overall decrease, our informants estimated that there has been over the last decades a clear upward trend in the number of international health volunteers working with humanitarian agencies, while the number working with the category of development organizations has strongly decreased. VSO and UNV did not report such important changes over time, but both report that recently there is a growing interest from recipient countries for medically qualified volunteers.

Some organizations reported important changes over the last two decades. Most notably the younger, secular medical NGOs, such as Médecins Sans Frontières, Handicap International and Action Contre La Faim, have grown fast. Other organizations, such as Cordaid and Medicus Mundi reported very steep decreases.

### Financial aspects

The lowest costs were reported by organizations such as Doctors without Vacation, that work essentially with short-term volunteers who do not receive any allowance. The cost for one mission is estimated at US$2400 per person, exclusively for travel and housing. Missions typically take two to three weeks.

Agencies sending volunteers for longer periods typically pay fees or allowances, raising the total annual cost to typically between US$36 000 and US$50 000 (range US$26 000 – 60 000).

Several organizations report that cost is largely independent of qualification and experience, as these are often not taken into account for the level of allowance, or only to a limited extent. It should be noted that these estimates could hide subsidies, such as social security contributions – which may be directly covered by the government – or accommodation, which is sometimes covered by the host institution.

### The perspective of volunteer organizations

We encountered a wide diversity of opinions among volunteer organizations regarding the role of international health volunteers. Different objectives were mentioned: 'covering humanitarian needs'; 'catalyst for change'; 'introduction of innovation'; 'capacity building'; 'project management' or 'personal solidarity'; 'link between North and South'. In fact, the choice of many NGOs to work in certain countries or regions is determined to a large extent by the fact whether this country is in crisis or in a process of post-conflict, such as is the case in Liberia, Sierra Leone. Most organizations do not see the sending of international health volunteers as a quantitative or gap-filling measure in countries with HRH shortages. Only a few organizations, in particular Voluntary Service Overseas-UK [5] and UN Volunteers, are at present explicitly increasing the number of international health volunteers to palliate HRH shortages in some low-income countries.

As was noted above, several organizations are reducing the number of international health volunteers, or even stopping to send any altogether. This is influenced by several factors. First, changes in thinking about development, where establishing long-term relations with partners, capacity building and recruitment of local staff gets the priority [6,7]. Second, the policy of certain donor governments may have contributed to this. For instance the Dutch government traditionally subsidized deployment of international health volunteers, but now discourages this by reducing budgets for expatriation programmes. Similar evolutions have taken place in Scandinavian countries and in Belgium. An important factor is the difficulty reported by a number of organizations to recruit medically qualified volunteers in their home societies in Europe and North America. It was also reported that many volunteers from the North prefer short contracts of a few months, after which people may or may not leave again for subsequent contracts. This preference results in a high staff turn over, and 'hopping' or 'shopping' between volunteer organizations.

In reaction to reduced attraction of expatriate work, some organizations said that they are progressively more recruiting from low-income countries, such as the Philippines, India, Bangladesh, Democratic Republic of Congo and Ethiopia. In most organizations however, these professionals from low-income countries still constitute a minority. Organizations expressed mixed feelings about such recruitment, as sometimes this is felt as contributing to the brain drain from these countries. Recruiting international volunteers from low-income countries is not cheaper than from high-income countries, but conditions offered are relatively more attractive for them as compared to other career options, thus facilitating recruitment.

NGOs from the industrialized world are also becoming important employers of health personnel in low-income countries. Indeed, in many organizations national doctors and nurses on their payroll now largely outnumber the expatriate volunteers.

### The perspective of country experts: a need for some nuance

During the group discussions, the country experts expressed a variety of views. In general, it seemed considerably easier to find weaknesses and negative views on the role of international health volunteers than strengths and positive experiences.

### Weaknesses

The view dominated that international health volunteers are mostly junior, inexperienced and ill prepared to work in low-income countries and this both for cultural and professional reasons. Examples abounded of young expatriates having difficulties with cultural and language barriers, and with differences in norms and values, resulting from insufficient cultural sensitivity and awareness. This was often compounded by important differences in lifestyles and living standards between expatriate volunteers and local colleagues, sometimes fuelling resentment.

There also was a shared perception that expatriate volunteers are too unfamiliar with local epidemiology, the local practice of health care and the organization of the health system. They were often seen to have insufficient technical skills, training and professional experience to work in their new environment. Quite often they were seen as undervaluing local staff knowledge. These problems are especially disturbing if volunteers come for short assignments, resulting in high turn over and lack of continuity.

The view was also expressed that expatriate volunteers often are unwilling to support the public health system, resulting from a lack of understanding of their role and lack of communication on their terms of reference, job description and mutual expectations. A different attitude to authority was also mentioned, resulting in the expatriate's inability or unwillingness to fit in the system and report to local managers. This results frequently in power struggles and conflicts with authorities. Not surprisingly, expatriate volunteers are often seen as highly focused on particular issues such as emergencies and AIDS, with little contribution to general health services. Moreover, they often prefer to create new parallel systems and procedures rather than supporting or improving the existing ones (e.g. assistance to refugees, creating tensions within the host population).

There was a widespread opinion that considers the presence of expatriate volunteers as paradoxical in view of the existence of urban unemployed doctors and nurses, with the exception of countries like Malawi, Mozambique and Zambia.

### Strengths

Most country experts had some experience with hard working, highly motivated and committed expatriate volunteers, who were willing to live and work in remote areas. These were then known as being inspiring and motivating for local staff, and often highly involved with local communities.

Such positive experiences were often seen with volunteers staying for longer periods of time, going through language training and investing initially in appropriate technical training, such as tropical medicine, epidemiology and health services organization. Such commitments were often accompanied by an influx of resources (funds, drugs and equipment), resulting not only in improved coverage of health services in underserved areas, but also in improved working conditions for local staff and real capacity building.

Other positive experiences with international health volunteers that were mentioned are:

▪ Willingness and/or ability of certain expatriate volunteers to work in difficult conditions (regions with political unrest or in post-conflict), where local health staff are unable or unwilling to work;

▪ Capacity to innovate, e.g. the creation of specific health programmes, such as antiretroviral therapy;

▪ Transfer of specific technical skills, especially by highly qualified expatriate consultants on short missions doing on-the-job training and bedside teaching;

▪ Strong management (including infrastructure) capacity of certain expatriates;

▪ Improved quality of teaching in educational institutions.

Most informants agreed that the presence and significance of international health volunteers extended well beyond their contribution to service delivery. They also viewed them as a concrete expression of international solidarity, international human relations, and cultural exchange. Moreover, they recognized the contribution of international health volunteers as advocates in their home society, ensuring public support for international solidarity and development aid in donor countries. Increased and better donor aid was viewed as crucial for improvement of health service delivery in sub-Saharan Africa.

### International health volunteers, an imposed solution?

When asked whether and under which conditions international health volunteers could positively contribute to filling the HRH gap in sub-Saharan countries, the consensus was that international health volunteers are a solution proposed by the North, which was not a high priority from their perspective.

The country experts thought that there was only a very limited place for international health volunteers in tackling the HRH crisis, if any. This was argued mainly in terms of cost-effectiveness and opportunity cost. There was a consensus that expatriate volunteers are costly, and considerably less cost-effective than locally hired staff. The majority of informants were strongly affirmative about the existence in their country of a considerable pool of health workers, who where unemployed or sub-employed in the capital, and that several of them could be readily recruited and motivated to work in under-served areas for the cost of one single expatriate volunteer. They roughly estimated that with the costs related to one expatriate, one could hire ten junior health workers.

Furthermore, our informants felt that recruiting expatriate volunteers while maintaining a recruitment stop for national health personnel was a real contradiction that needed to be exposed. Similarly, the co-existence of the brain drain of African doctors and nurses to the North with programmes to recruit young volunteers in the North to work in sub-Saharan Africa was seen as a paradox. Moreover, the brain drain out of the continent was considered many times more important quantitatively than the number of international volunteers.

### Alternatives

Participants proposed to focus more on the alternatives that in their opinion are insufficiently used.

In the relatively few countries where certain categories of health workers are not available, our informants would give priority to investment in increased training capacity to tackle HRH shortages more structurally in the longer term. The alternative of recruiting foreign doctors in government service, be they from Cuba, Congo or Nigeria, was also mentioned, but strengths and weaknesses of this option were not explored further.

Many informants also held the opinion that improving working conditions for national health personnel – by topping up salaries, improved supplies and equipment, and upgrading facilities – would enhance staff productivity considerably, and go a long way in palliating present staff shortages.

In countries where certain categories of staff are critically lacking (e.g. doctors in Malawi, Zambia, Mozambique or Zimbabwe), the informants saw a possible place for expatriate volunteers to palliate such critical staff shortages in government facilities or health training institutions, especially in under-served provinces.

As conditions for success, they would formulate the following:

▪ Clear identification of specific HRH needs prior to recruitment of international volunteers;

▪ Preference for experienced teachers and clinicians, aiming at transfer of knowledge and skills;

▪ For younger professionals, adequate training and preparation were considered essential, and attachment to local experienced health professionals during the first months of their assignment was considered very beneficial;

▪ Selecting only people who are prepared to work in a new cultural and organizational environment; and who accept to work within the local structures, complying with local rules and regulations, and respecting local lines of authority; and

▪ Recruiting volunteers for a significant duration of stay (three to five years were mentioned, except for certain specific technical specialists where shorter periods could be useful, especially when repeated at regular intervals).

## Discussion

### Notes on methodology and data collection

Before we discuss the limitations of this study, it should be noted that we focused on the larger European organizations and United Nations Volunteers (UNV). Although we had some telephone conversations with them, we do not report on organizations such as the Red Cross movement, Peace Corps-USA, Save the Children-USA, Care USA, Mission Doctors Association USA, World Vision USA, Rotary Doctor's Bank and many of the smaller NGOs. Bilateral or multilateral organizations, the International Committee of the Red Cross or staff directly recruited by governments were not included, as we did not consider it to be volunteer organizations.

Some limitations need to be taken into account. First, access to data was not easy. Medical NGOs such as Handicap International or Médecins du Monde are organized as a network of relatively independent national organizations. Their international secretariats often cannot provide aggregated data on human resources deployed by the national branches. We then focused on the most important agency, usually the 'mother house'. In practice, this means that we obtained relatively little information on the total number of volunteers sent by the smaller organizations and by the Christian missionary organizations. The group discussions confirmed that mission hospitals employ expatriate medical staff in most countries of sub-Saharan Africa, but we found little information on their quantitative importance from the survey among organizations.

Second, some organizations could not provide us with an estimated number of full-time positions. Consequently, the total number of international health volunteers reported is a mix of prevalence and incidence data, which makes it more difficult to compare. In an attempt to make the data somehow comparable across organizations, we estimated the full-time equivalent positions in sub-Saharan Africa for international health volunteers.

### A significant but small contribution

Our survey shows that at any point in time in 2005, around 2072 international health volunteers were deployed in sub-Saharan Africa by the larger volunteer organisation (Table). The limitations discussed above may lead to an underestimation of the number of expatriate staff deployed. However, the most likely source of underestimation would be the many small organizations that send out less than 10 volunteers. We would, therefore, be surprised if the total number were to reach more than 5000, and we venture to put forward this number as a ceiling. Between 25% and 30% of these are medical doctors. We estimate therefore that there are a grand maximum of 1500 expatriate volunteer doctors working in sub-Saharan Africa. The number of international health volunteers working in sub-Saharan Africa is thus relatively limited, as compared to the estimated HRH gap in the continent, which is estimated in the hundreds of thousands [8]. These numbers are insignificant indeed when compared with the more than 20 000 Cuban doctors working in Venezuela.

Moreover, we have the impression that the total numbers for all agencies combined have been decreasing over the last decades. We could not obtain hard data on this, but this is strengthened by converging anecdotal information from umbrella volunteer organizations, from training courses in tropical medicine and from recipient countries. Over the same period, most countries in sub-Saharan Africa have considerably increased their own medical workforce. This fits in the larger picture, where the wide awareness about the HRH crisis in sub-Saharan Africa is relatively new [8], and where the situation of an absolute HRH shortage is limited to a few countries (e.g. Mozambique, Malawi [9,10], Zambia [11], and Rwanda [10]) where it threatens service delivery or roll out of new programmes. This is illustrated by a study on medical doctors in Zambia [12], which shows that Zambia has only 632 medical doctors working in government and church services, 245 of whom are foreigners. Among them not more than 20 to 30 are employed by volunteer organizations, while 120 are from other African countries, directly employed by the Zambian government or Zambian health facilities. So, even in countries with a severe doctor shortage, such as Zambia, expatriate volunteer doctors only represent a relatively small proportion of the overall number of doctors, even of the expatriate doctors. However, where they work, be it in government or mission health facilities, they often play a crucial role, especially in underserved provinces. Sending 20 or 50 extra volunteer doctors to such a country could make an important difference for health service delivery.

Also the contribution of Peace Corps (US), which reported that 1500 of their volunteers worked in health and HIV/AIDS projects worldwide but very few in clinical work, is in line with our findings on the limited quantitative contribution of international health volunteers in health service delivery in sub-Saharan Africa.

Finally, anecdotal evidence from Zambia, Zimbabwe, Botswana, South Africa and Mozambique reveals that there are sizable contingents of expatriate doctors in these countries employed, especially from Cuba, Nigeria and the Democratic Republic of Congo. In these countries, their numbers are considerably higher than those of expatriate doctors employed by Western volunteer organizations, often 10 times higher.

### Deployment profiles of volunteer organizations

Humanitarian organizations often work in emergencies and crisis situations, or focus on AIDS projects. They represent over half of all expatriate health volunteers we could document in our survey, with Médecins Sans Frontières by far the largest contributor. Their recent growth is explained by a number of factors, but they do not aim at systematic gap filling for HRH shortages, certainly not in government health services. Country experts do not perceive them as having that potential either. Their role is seen as focused on short-term projects, which are not the primary concern of government policy makers, who are more focused on staffing government health facilities.

Our informed impression is that development NGOs, especially those rooted in faith-based missionary organizations, have been drastically scaling-down the number of expatriate doctors and nurses they send to sub-Saharan Africa. In most countries, local staff has taken over the tasks previously assumed by expatriates, also in mission hospitals, as documented by Cordaid in Ghana [6]. However, in the countries with a limited health workforce, also mission hospitals face difficulties retaining their workforce (e.g. in Uganda), while the importance of their contribution to health service delivery is widely acknowledged. It could be explored whether these organizations would be willing and able to reverse the declining trend in expatriate recruitment, and again supply larger numbers of expatriate health workers to countries with a serious HRH deficit. This could contribute to maintaining and expanding service delivery in missionary health facilities now that demand for care is fast increasing, mainly due to the impact of AIDS.

Volunteer organizations such as VSO and UNV have recently been responding to requests from recipient countries to increase recruitment of expatriate medical volunteers. They may be able and willing to recruit more, probably hundreds rather than thousands, be it in the North or in other middle- or low-income countries. The U.S. Global Health Service Corps plans to initially recruit 150 professionals.

### Cost

Volunteer organizations estimate the cost of posting an expatriate volunteer to be most often between US$36 000 and US$50 000 per year. This cost does not vary greatly with qualifications or experience, nor with geographical origin of volunteers. The total cost of the estimated maximum of 5000 international health volunteers in sub-Saharan Africa would then amount to between US$180 million and US$250 million annually.

### Country perspective

#### Which role for international health volunteers?

Strikingly, the views expressed in the discussion groups appeared inconsistent and contradictory, until it became clear that country experts identify relatively distinct types of expatriate volunteers in sub-Saharan Africa, with quite different strengths and weaknesses.

Many of the weaknesses and criticisms were directed towards the NGO volunteers working in NGO projects, who were perceived as mostly young and inexperienced, ill-prepared, staying too short a time, and engaged in highly focused activities that often did not fit in with the overall national health policy.

Much greater appreciation was reserved for expatriate volunteers working in mission hospitals, or for those seconded by volunteer agencies to government facilities. Both these categories were perceived as fitting well within – and strengthening – existing structures and having more appropriate qualifications. They were also seen as benefiting from better coaching and usually longer-term commitments. However, also short-term missions of appropriately chosen senior consultants were perceived as generally positive.

The country experts made a distinction among international health volunteers in three categories. The categories may not exactly coincide with the categories of volunteer organizations sending the volunteers, but are somehow similar. However, despite this nuanced appreciation of different categories of expatriate volunteers, the informants had in general strong reservations against relying on international volunteers to tackle the HRH crisis in their countries. Their opinion is very similar to the concerns expressed regarding international volunteers in a background document for the High-Level Forum on the Health MDGs held in Abuja in December 2004 [4]. This document states that the overall cost of bringing in expatriate volunteers compares unfavourably with the cost of retention measures for national health workers, and that relying on such volunteers may carry the risk of postponing critical decisions on pay and incentives for the national workforce. The document also concludes that international volunteers can be considered for gap filling in peripheral service delivery, with a preference for southern international volunteers, but only as a last resort measure, or supplementary measure where other measures fail to create the necessary response to the HRH crisis. The recent experience in Zambia, making the shift from a supplementation programme of Dutch medical doctors to a retention scheme for Zambian medical doctors lends some support to this view [12]. However, it should be noted that the serious doctor shortage remains and current measures seem unable to fundamentally reverse the trend [13].

## Conclusion

The quantitative contribution of international health volunteers to the health workforce in sub-Saharan Africa is at present limited and probably decreasing. The relative share of humanitarian NGOs among expatriate health volunteers is increasing, while they play a limited role in HRH gap filling. The number of international health volunteers sent by development-oriented NGOs, mainly to mission hospitals, seems to be drastically decreasing. Only a few agencies, especially Voluntary Service Overseas and United Nations Volunteers, seem prepared to increase their recruitment of expatriate health volunteers, and a few of the countries with the most severe HRH crisis may be asking for such support. However, country health service managers in sub-Saharan Africa consider international volunteers as a last resort measure, judging that it is not very cost-effective, as compared with investment in local capacity.

It is our impression that in a limited number of countries in Southern and Eastern Africa, which combine a high burden of HIV/AIDS with critical HRH shortages, the reliance on international health volunteers is likely to increase over the coming years, especially for expatriate doctors. Some of these countries indeed face decreasing numbers of doctors for health service delivery at the time they start to scale-up access to antiretroviral treatment, which is very labour intensive. Both government and mission hospitals may be facing critical shortages, especially of medical doctors. UNV, VSO and the new U.S. Global Health Service Corps are prime candidates as volunteer agencies for sending these volunteers. However, the numbers involved are likely to remain relatively limited.

Moreover, countries are likely to be very alert to the cost of such initiatives and to compare them with other strategies to strengthen their own medical workforce, or to hire expatriate doctors in government service themselves.

However, all actors interviewed stressed that the role and significance of expatriate health volunteers is much broader than their quantitative contribution to the health workforce in sub-Saharan Africa. From their different perspectives, most informants – also those representing the views of African government officials – had good reasons to defend the continued presence of expatriate health volunteers in a variety of situations and roles.

In summary, our survey reveals that on the whole the present contribution of international health volunteers to the health workforce is rather limited, even in countries facing a severe HRH crisis. It seems also that only in exceptional circumstances their contribution can be considerably increased, but in these exceptional circumstances their role may be very significant.

## Competing interests

The author(s) declare that they have no competing interests.

## Authors' contributions

GL made a substantial contribution to the conception, design, acquisition as well as analysis and interpretation of results. He was also involved in drafting the manuscript. GK, BM and DVDR made contributions to the conception of the research and revising the intellectual content of the paper. IB collected data and reviewed successive versions of the paper. WVD made substantial contribution to the conception, design as well as analysis and interpretation of results. He was responsible for drafting successive versions of the manuscript.
